# Impact of a prospective feedback loop aimed at reducing non-beneficial treatments in older people admitted to hospital and potentially nearing the end of life. A cluster stepped-wedge randomised controlled trial

**DOI:** 10.1093/ageing/afae115

**Published:** 2024-06-09

**Authors:** Nicole M White, Adrian G Barnett, Xing J Lee, Alison Farrington, Hannah Carter, Steven M McPhail, Magnolia Cardona, Kenneth Hillman, Leonie Callaway, Lindy Willmott, Ben P White, Gillian Harvey, Nicholas Graves, Christine Brown

**Affiliations:** Australian Centre for Health Services Innovation and Centre for Healthcare Transformation, School of Public Health and Social Work, Faculty of Health, Queensland University of Technology, Kelvin Grove, Queensland, Australia; Australian Centre for Health Services Innovation and Centre for Healthcare Transformation, School of Public Health and Social Work, Faculty of Health, Queensland University of Technology, Kelvin Grove, Queensland, Australia; Australian Centre for Health Services Innovation and Centre for Healthcare Transformation, School of Public Health and Social Work, Faculty of Health, Queensland University of Technology, Kelvin Grove, Queensland, Australia; Australian Centre for Health Services Innovation and Centre for Healthcare Transformation, School of Public Health and Social Work, Faculty of Health, Queensland University of Technology, Kelvin Grove, Queensland, Australia; Australian Centre for Health Services Innovation and Centre for Healthcare Transformation, School of Public Health and Social Work, Faculty of Health, Queensland University of Technology, Kelvin Grove, Queensland, Australia; Australian Centre for Health Services Innovation and Centre for Healthcare Transformation, School of Public Health and Social Work, Faculty of Health, Queensland University of Technology, Kelvin Grove, Queensland, Australia; Digital Health and Informatics Directorate, Metro South Health, Woolloongabba, Brisbane, Australia; Bond University Evidence Based Practice Professorial Unit, Gold Coast University Hospital, Southport, Queensland, Australia; School of Population Health, The University of New South Wales, Kensington, New South Wales, Australia; Simpson Centre for Health Services Research, South West Sydney Clinical School, University of New South Wales, Liverpool, New South Wales, Australia; School of Clinical Medicine, University of New South Wales, Liverpool, New South Wales, Australia; Ingham Institute for Applied Medial Research, Liverpool, New South Wales, Australia; Royal Brisbane and Women’s Hospital, Herston, Queensland, Australia; Faculty of Health, Queensland University of Technology, Kelvin Grove, Queensland, Australia; Faculty of Medicine, University of Queensland, Herston, Queensland, Australia; Australian Centre for Health Law Research, School of Law, Faculty of Business and Law, Queensland University of Technology, Brisbane, Queensland, Australia; Australian Centre for Health Law Research, School of Law, Faculty of Business and Law, Queensland University of Technology, Brisbane, Queensland, Australia; Australian Centre for Health Services Innovation and Centre for Healthcare Transformation, School of Public Health and Social Work, Faculty of Health, Queensland University of Technology, Kelvin Grove, Queensland, Australia; College of Nursing and Health Sciences, Flinders University, Bedford Park, South Australia, Australia; Australian Centre for Health Services Innovation and Centre for Healthcare Transformation, School of Public Health and Social Work, Faculty of Health, Queensland University of Technology, Kelvin Grove, Queensland, Australia; Duke-NUS Postgraduate Medical School, National University of Singapore, Singapore; Australian Centre for Health Services Innovation and Centre for Healthcare Transformation, School of Public Health and Social Work, Faculty of Health, Queensland University of Technology, Kelvin Grove, Queensland, Australia

**Keywords:** non-beneficial treatment, stepped-wedge trial, advance care planning, older people, end of life

## Abstract

**Objectives:**

To investigate if a prospective feedback loop that flags older patients at risk of death can reduce non-beneficial treatment at end of life.

**Design:**

Prospective stepped-wedge cluster randomised trial with usual care and intervention phases.

**Setting:**

Three large tertiary public hospitals in south-east Queensland, Australia.

**Participants:**

14 clinical teams were recruited across the three hospitals. Teams were recruited based on a consistent history of admitting patients aged 75+ years, and needed a nominated lead specialist consultant. Under the care of these teams, there were 4,268 patients (median age 84 years) who were potentially near the end of life and flagged at risk of non-beneficial treatment.

**Intervention:**

The intervention notified clinicians of patients under their care determined as at-risk of non-beneficial treatment. There were two notification flags: a real-time notification and an email sent to clinicians about the at-risk patients at the end of each screening day. The nudge intervention ran for 16–35 weeks across the three hospitals.

**Main outcome measures:**

The primary outcome was the proportion of patients with one or more intensive care unit (ICU) admissions. The secondary outcomes examined times from patients being flagged at-risk.

**Results:**

There was no improvement in the primary outcome of reduced ICU admissions (mean probability difference [intervention minus usual care] = −0.01, 95% confidence interval −0.08 to 0.01). There were no differences for the times to death, discharge, or medical emergency call. There was a reduction in the probability of re-admission to hospital during the intervention phase (mean probability difference −0.08, 95% confidence interval −0.13 to −0.03).

**Conclusions:**

This nudge intervention was not sufficient to reduce the trial’s non-beneficial treatment outcomes in older hospital patients.

**Trial registration:**

Australia New Zealand Clinical Trial Registry, ACTRN12619000675123 (registered 6 May 2019).

## Key Points

- Most patients were found to be nearing the end of their life.
- A nudge intervention did not change patient management.
- More directive interventions and policy changes may be needed to reduce non-beneficial treatment.

## Introduction

For patients nearing the end of life, the benefits of treatment with curative intent can be greatly outweighed by the treatments’ harms. A focus on symptom control, maximising function, quality of life and focusing on what matters to the patient may be a better approach to care. Most expected deaths in high-income countries occur in acute hospitals [1] suggesting that hospital-based medical teams are an essential in providing optimal end-of-life care. However, clinicians continue to identify barriers to providing optimal end-of-life care to their patients, citing difficulty in withdrawing curative care because of their strong training in curative care and prognostic uncertainty [2], lack of training in end-of-life care, time constraints, legal concerns and the potentially contradictory wishes of patients and their families [3, 4]. This situation means that many older patients in high-income countries continue to experience non-beneficial treatment at the end of life.

Non-beneficial treatment is also referred to as ‘futile treatment’, ‘potentially inappropriate treatment’ or ‘over-treatment’. Receiving non-beneficial treatment can be painful, distressing for patients and their families and costly for the health system [5, 6]. Most Australians do not want to die in hospital [7, 8], and avoiding highly medicalised deaths would accord with most patients’ preferences [9]. A large Australian observational study of in-hospital deaths found the median time between recognition of dying to death was just 2 days, and half of all patients received active interventions in their last 48 hours of life [8]. A previous systematic review of international studies found that 33–38% of patients received non-beneficial treatment at the end of life [10]. Research into improving end-of-life care is therefore a significant global issue unchanged over the last decade.

The reasons why non-beneficial treatments are provided at the end of life are complex and arise from clinician factors, patient factors and hospital factors [3, 4, 11, 12]. The difficult but necessary conversations with patients around goals of care require high-level clinical skills and experience [4, 13], and discussions can be difficult even with older patients who are ready to die [9]. Avoiding these conversations and not putting care plans in place means that hospitalised older patients can be poorly prepared for their approaching death [14] and are at-risk of experiencing unnecessary suffering. Many patients would have had a better death in a palliative care setting where they could say goodbye to their loved ones and have their pain managed. The recent *Lancet* Commission on the Value of Death identified the need to change the mindset and rules of healthcare systems, so that death ‘transcends’ healthcare and that healthcare services are accountable for their relief of suffering and management of good deaths [15].

### The Intervention for Appropriate Care and Treatment trial

The primary outcome measure of the Intervention for Appropriate Care and Treatment (InterACT) trial was the proportion of patients with one or more ICU admissions.

Secondary outcomes of the trial were:

1. length of hospital stay and discharge outcome
2. time to hospital re-admission
3. time to first documented clinician-led care review discussion
4. time to care directive measures
5. time to palliative care referral
6. time to medical emergency call
7. changes in admission and treatment costs
8. cost of implementing the prospective feedback loop intervention
9. extent and fidelity of intervention implementation, impact and contextual barriers and enablers of the feedback loop intervention.

This paper reports the trial’s primary outcome and secondary outcomes 2, 3 and 7. We have previously reported on secondary outcomes 4, 5 and 6 using data immediately available on trial completion [16]. The cost-consequence analysis, outcomes 8 and 9, will be published separately, and the process evaluation results (outcome 10) are available [17]. ICU admission was chosen as the primary outcome due to the increasing number of ICU admissions of older patients [18] and the potential for highly invasive treatments that unnecessarily increase suffering at the end of life. An observational study on the end of life in Australian hospitals reviewed 1,692 in-hospital deaths, with 356 (21%) of these in ICU and 27% of these ICU deaths in patients 75 years and older [8]. Another Australian study of older people admitted to ICU found that 39% of patients aged 50 or over were frail and 19% of these patients died in hospital [19].

The secondary outcomes of length of hospital stay and readmission rates were hypothesised to decrease in the intervention phase as a result of an increase in care review outcomes (secondary outcomes 4, 5 and 6). A medical emergency team (MET) call is a hospital-based system to call senior staff, usually from the ICU for help when a patient's vital signs have fallen outside set criteria. It is also known as a rapid response team (RRT) call [20]. A MET call was chosen as an outcome as up to one-third of patients at end-of-life experience a MET call with the potential for futile resuscitation attempts [10, 21].

The results from outcomes 4, 5 and 6 were disappointing as an intervention designed to improve care of hospitalised older people appeared to have the opposite effect on care review outcomes [16]. The reasons for this may be a combination of the intervention design, further discussed in the process evaluation [17], and health system challenges due to the COVID-19 pandemic.

The InterACT trial aimed to test a prospective feedback loop in three acute hospitals to increase appropriate care and treatment decisions and pathways for older populations at the end of life.

The primary objectives were to:

- determine the impact and resource use and costs of a tailored clinical team feedback loop intervention on patient outcomes related to appropriate care and treatment at the end of life
- conduct a process evaluation to assess implementation, mechanisms of impact and contextual barriers and enablers of the feedback loop intervention

The intervention reported on here builds on an earlier study regarding why some doctors knowingly provide futile treatment to patients who are ‘at the end of life’ [3]. Accordingly, although a distinction can be made between ‘end of life’ and ‘at risk of death’, we have adopted the terminology that patients will be considered ‘at the end of life’ if they have been screened as ‘at risk of death’. This also reflects the intent of the screening tools and patient cohort (75 years or older).

## Methods

The trial study design and analyses were pre-specified in a protocol [22], and the trial was pre-registered (ACTRN12619000675123).

The study was a stepped-wedge cluster randomised trial design, conducted in three large public hospitals in south-east Queensland, Australia. We used a stepped-wedge design because the intervention was aimed at whole clinical teams rather than individual patients, making patient-level randomisation unsuitable. Each hospital was a cluster, where each cluster was randomised to its own treatment sequence. The number of teams per hospital was three, four and seven. A general medical ward was included in each hospital, alongside teams that specialised in cardiology, stroke, vascular, respiratory, nephrology, neurosurgery and orthopaedics.

The trial design is illustrated in Appendix 1. Each cluster-time period was equal to 1 week, which resulted in 75 cluster-periods in the usual care phase and 75 cluster-periods in the intervention phase. Each hospital started with a usual care phase to provide data on current practice and patient outcomes. A hospital’s usual care phase was followed by a 4-week implementation establishment phase, during which the changes to practice were discussed as well as potential tailoring of the intervention by clinical teams. Tailoring included the mechanisms of how at-risk patients would be flagged with the teams and an agreed clinical response. The intervention phase provided data on how the intervention changed patient care and outcomes.

At the start of the trial, hospitals were randomised to treatment sequences, where each treatment sequence defined the timing of the intervention. The treatment sequences were randomised by a statistician (XJL). Each hospital was treated as a cluster and randomly allocated to intervention timing prior to trial commencement. Hospitals were notified 8 weeks prior to the start of their intervention phase. Blinding of the intervention timing was not possible.

The COVID-19 pandemic greatly changed usual practice in the three hospitals, and it was not possible to embed non-essential study staff in the hospitals. This delayed the start of the trial by 8 weeks.

### Inclusion and exclusion criteria

The intervention was targeted at clinical teams who saw relatively large numbers of older (aged 75 and over) patients. Included clinical teams must have: been an established clinical team unit or specialty with a consistent history of admitting patients aged 75 years or over during the previous year; included at least one nominated lead specialist consultant; included at least one registrar and affiliated clinical nurse consultant or nurse unit manager; had a clinical team structure and admission pattern typical of the hospital; and participated in an information session with the project team.

Excluded clinical teams were those already implementing interventions or initiatives related to reducing non-beneficial treatments for older patients and teams from emergency departments, intensive care units (ICUs), mental health units and non-acute care, as these specialities would require a different implementation plan due to their different clinical and treatment focus.

### Ethical considerations

Clinical teams were identified by purposive sampling. Consent was provided by a nominated lead clinician.

The study received ethics approval for all participating hospitals from the Royal Brisbane and Women’s Hospital Human Research Ethics Committee (HREC) (Approval HREC/2019/QRBW/51606) and the QUT HREC (Approval 1900000630). These approvals included a waiver of consent for access to patient data and to health services data. A Public Health Act (PHA) application was approved for patient data linkage (RD008146). In accordance with the Queensland Health Research Governance requirements, site-specific assessment (SSA) approvals were obtained from the three hospitals (Gold Coast University Hospital, Royal Brisbane and Women’s Hospital, The Prince Charles Hospital).

### Identifying at-risk patients

All patients aged 75 or over who were admitted to the care of the included clinical teams were screened for being near the end of life and at risk of non-beneficial treatment. Patients were screened from May 2020 to June 2021. We used two published tools designed to identify patients towards the end of life: the Criteria for Screening and Triaging to Appropriate aLternative Care (CriSTAL) and the general indicators of the Supportive and Palliative Care Indicators Tool (SPICT™). The use of the SPICT was limited to the general indicators as the auditors did not have access to patients. We believe that the co-morbidities and other risk factors measured with in the CriSTAL tool overlapped with the clinical indicators and life-limiting conditions from the second section of the SPICT tool. CriSTAL was developed to identify older patients in the last three months of life [23] and SPICT™ to identify patients at risk of deteriorating and dying [24–26]. Patients with a CriSTAL score of 6 or greater or SPICT™ score of 2 or greater were classified as at-risk.

To identify the at-risk patients, hospital-based trained auditors reviewed patient records of new admissions to the clinical teams twice weekly. The auditors entered data using an electronic form in REDCap [27]. Screening occurred during the usual care and intervention phases.

Patient-level exposure and measurement under the trial design was characterised by continuous recruitment with short exposure [28]. This meant that patients were exposed to the intervention from the time they were identified as at-risk to the time the outcome of interest was experienced.

### Intervention

The Intervention for Appropriate Care and Treatment (InterACT) was a feedback intervention where at-risk patients were flagged with the clinical teams with the expectation of triggering a clinical response. The aim was to prompt actions that might reduce non-beneficial treatment, including reviews of care directive measures, clinician-led discussions, and palliative care referrals. The implementation was tailored by hospital as each hospital had their own systems. The notification system in each hospital was either an orange flag next to the patient’s name on the electronic patient journey board, information in the medical handover report, or an alert in a patient’s medical record.

Clinical teams could opt in to receiving automated emails about at-risk patients that showed the elements of the CriSTAL and SPICT™ tools. An example report is shown in Appendix 2.

### Outcomes

Our hypothesis was that the intervention would result in a different hospital experience for patients, with fewer non-beneficial treatments and reduced morbidity.

The primary outcome was one or more ICU admissions during the current hospital admission from the date first recorded as at-risk. Admission to an ICU is considered a major intervention reserved for patients with an acceptable prognosis.

The secondary outcomes examined here were hospital length of stay and patient outcome (discharge or death), time from at-risk identification to first medical emergency team call and time from discharge to hospital re-admission within 12 weeks from discharge. These outcomes were deemed as important aspects of patient experience and indicators of potential non-beneficial treatment. Extended hospital stays, potentially ending in a protracted death, can be extremely distressing for patients and families [15]. Medical emergency team calls and re-admissions to hospital soon after discharge can signify that a patient has not been recognised as close to death and can result in unwanted treatments such as cardiopulmonary resuscitation.

### Statistical methods

The target sample size of 3,870 at-risk patients was estimated to give a statistical power of 95% to detect a reduction in the proportion of at-risk patients with ICU admissions from 0.20 in the usual care phase to 0.113 in the intervention phase. This calculation used a two-sided 5% statistical significance level, intraclass correlation of 0.1 and the stepped-wedge design assuming an exchangeable correlation structure.

The primary outcome of ICU admissions was modelled as a binary variable in a regression model. The key independent variable was the study phase (usual care or intervention phase). We included calendar time to model a potential linear trend [29] and adjusted for the patients’ age and gender. Calendar time was defined as the number of weeks since the start of the trial, based on patients’ date of hospital admission. The model included a random intercept for each clinical team to account for correlated data from the same team due to similarities in the proportion of patients with ICU admissions, for example, a team that generally dealt with more complex patients.

The secondary outcomes were modelled using time-to-event analysis. Hospital length of stay was modelled with death and discharge as competing risks [30]. All models were adjusted for patient age and gender, and clinical team was defined as strata. Results were summarised as relative hazard ratios and absolute risk differences at 90 days, which was chosen as the CriSTAL at-risk tool is for death within 3 months [31].

In a sensitivity analysis, we accounted for seasonal changes in the outcomes as, despite the subtropical climate, older people in south-east Queensland are often impacted by influenza and low temperatures [32, 33]. Seasonality was calculated using the patients’ hospital admission dates.

In a subgroup analysis, we examined the results by hospital, as the effectiveness of health system interventions can vary by hospital due to the importance of local factors.

All data management and analyses were conducted using R version 4.1.0. The code and synthetic data [34] are available online here: https://github.com/agbarnett/InterACT, and the full data can be requested using the QCIF dataverse: https://doi.org/10.60540/PT6IPY.

The results are reported using the CONSORT extension for stepped-wedge cluster-randomised trials [35]. We report the results blinded to hospital.

Details on the statistical methods are in Appendix 3.

### Patient and public involvement

Feedback from key stakeholders was used to refine the intervention. We used a three-person health consumer group convened with Health Consumers Queensland to get feedback on the acceptability of the intervention to patients and families. We used focus groups with clinicians to get their feedback on the criteria used to flag at-risk patients and how the intervention would be implemented.

## Results

**Figure 1:**
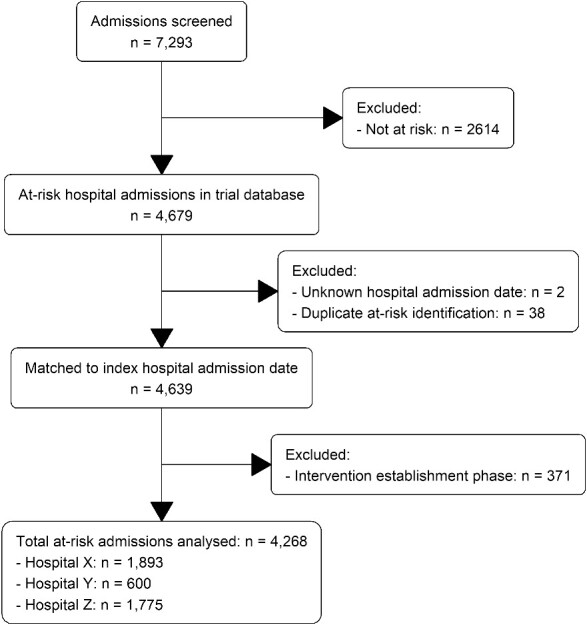
Flow chart of the number of admissions screened and included in the analysis.

A flow chart of included patients is shown in Figure 1.

There were 4,268 admissions where the patient was estimated to be at-risk of non-beneficial treatment, with 2,142 in the usual care phase and 2,126 in the intervention phase. This exceeded our target sample size by 10%. Patients were screened between 25 May 2020 and 6 June 2021 (378 days). The patients’ characteristics by phase are summarised in Table 1, showing no clear differences in their overall characteristics between the usual care and intervention phases. Data on positive COVID-19 infection were collected as part of the screening process; however, COVID-19 was not an issue in this sample with only four positive tests amongst the over 8,000 patients screened.

**Table 1:** Descriptive table of included patient admissions for the usual care and intervention phases. Q1–Q3 is the first to third quartile

	Usual care	Intervention
Weeks	75	75
Number at risk	2,142	2,126
Admission dates, median [Q1–Q3]	Aug 2020 [Jul 2020 to Oct 2020]	Feb 2021 [Dec 2020 to Apr 2021]
Female, *n* (%)	52%	56%
Age, median [Q1–Q3]	84 [79–88]	84 [79–89]
CriSTAL score, median [Q1–Q3]	5 [4–6]	5 [4–6]
SPICT score, median [Q1–Q3]	2 [2–3]	3 [2–3]
Admitted from nursing home or supported accommodation, %	26%	23%
Admitted via emergency department, %	92%	93%

**Figure 2:**
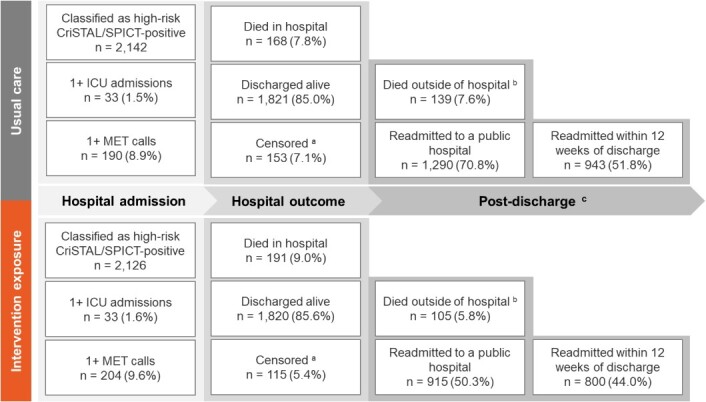
Number and percentages of outcomes across the patient’s journey from admission to post-discharge. Results for all three hospitals combined. (a) includes patients who were still in hospital during the transition from usual care to intervention phase, or at the end of the study; patients transferred from the admitting study hospital to another facility for continuing care, (b) includes patients who died without readmission to hospital, and (c) includes deaths and readmissions to hospital greater than 12 weeks post-discharge. The total numbers of discharged patients without a readmission and who were alive at the end of the trial were *n* = 392 during usual care and *n* = 800 during the intervention phase.

The primary and secondary outcome numbers are summarised in Figure 2, which highlights how the outcomes were staged across the patient journey. For example, re-admissions to hospital were only possible in those patients who were discharged.

There was no difference in the primary outcome of the proportion of patients with one or more ICU admissions, with an odds ratio close to 1 and small difference in probability (Table 2).

**Table 2:** Estimated effect of the intervention, results with and without adjusting for season. We give relative estimates using ratios and absolute estimates using probabilities. Ratios below 1 indicate a reduced odds or hazard (longer times) in the intervention phase compared with usual care, whilst those above 1 indicate an increased odds or hazard (shorter times). Probabilities above zero indicate an increase in the intervention phase compared with usual care

		Not adjusted for season	Adjusted for season
Relative estimates using ratios (intervention/usual care)			
Outcome	Estimate	Mean and 95% CI	Mean and 95% CI
ICU admission	Odds ratio	0.84 (0.33–2.16)	0.70 (0.26–1.90)
Time to discharged alive	Hazard ratio	1.01 (0.94–1.08)	0.90 (0.81–0.99)
Time to death in hospital	Hazard ratio	1.36 (1.06–1.75)	0.88 (0.61–1.26)
Time to medical emergency call	Hazard ratio	0.93 (0.75–1.15)	0.94 (0.67–1.31)
Time to hospital re-admission	Hazard ratio	0.84 (0.76–0.93)	0.74 (0.63–0.86)
Absolute estimates using probabilities (intervention minus usual care)			
Outcome	Estimate	Mean and 95% CI	Mean and 95% CI
ICU admission	Mean difference	−0.01 (−0.07 to 0.02)	−0.01 (−0.08 to 0.01)
Discharged alive	Mean difference at 90 days	0.000 (−0.002 to 0.002)	0.001 (−0.002 to 0.005)
Death in hospital	Mean difference at 90 days	0.07 (0.01 to 0.14)	0.03 (−0.06 to 0.13)
Medical emergency call	Mean difference at 90 days	−0.01 (−0.06 to 0.04)	−0.00 (−0.07 to 0.07)
Hospital re-admission	Mean difference at 12 weeks	−0.06 (−0.09 to −0.02)	−0.08 (−0.13 to −0.03)

The hazard of discharge was similar in the intervention and usual care phases (Table 2), but the hazard of death was increased during the intervention phase, indicating a generally faster time to death during the intervention period. There was also an absolute increase in the probability of death at 90 days of 0.07 (95% CI: 0.01 to 0.14) (Table 2). However, this increase in death probability was much smaller after adjusting for season (0.03; −0.06 to 0.13).

There was no difference in the time to medical emergency team calls (Table 2), with relatively narrow confidence intervals consistent with only small to moderate changes associated with the intervention.

The intervention phase was associated with a reduced hazard of hospital readmission (Table 2), suggesting a potential benefit. The absolute difference in probability at 90 days was a 0.06 reduction in readmission at 90 days in the intervention phase compared with usual care (95% CI: −0.09 to −0.02). This reduction was maintained after adjusting for season.

**Figure 3:**
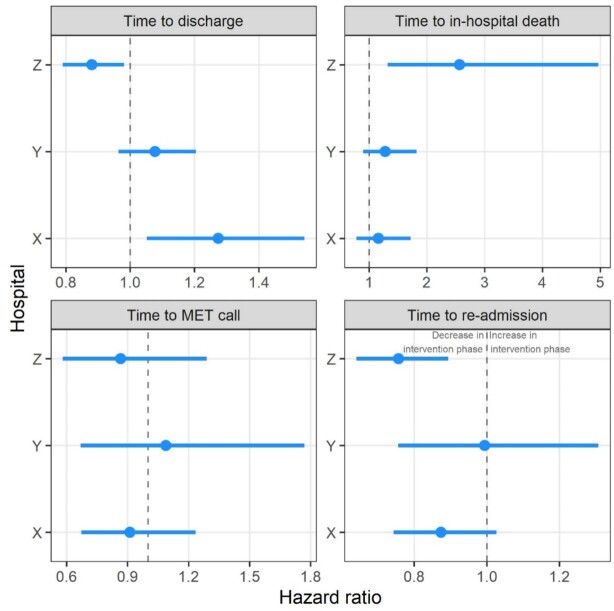
Mean hazard ratios (dots) and 95% confidence intervals (horizontal lines) by hospital for the time-to-event outcomes. The hazard ratio scales vary by outcome. MET, medical emergency team.

The secondary outcomes by hospital are summarised in Figure 3. The most heterogeneous result was for time to hospital discharge, where the intervention was associated with a reduced time to discharge in Hospital X and increased time to discharge in Hospital Z. The increased hazard for time to death was larger in Hospital Z.

**Figure 4:**
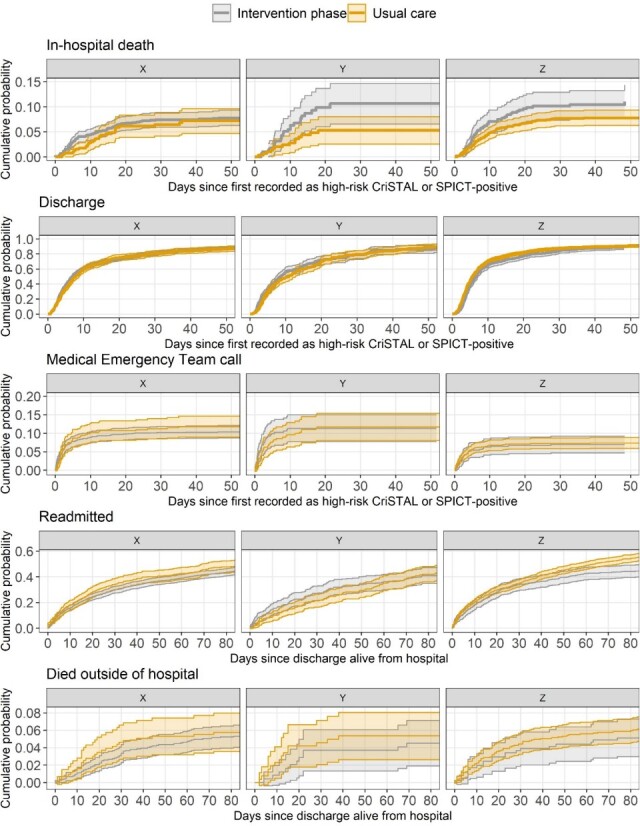
Cumulative probability curves for the time-to-event outcomes by hospital. Curves are given for the intervention and usual care phases. The shaded areas are 95% confidence intervals. The time axes for the top three panels end at 50 days and for the bottom two panels at 80 days as there were few events after these times.

Cumulative probability curves for the time-to-event outcomes by hospital are shown in Figure 4, which show the accumulation of outcomes over time. Most of the curves show an overlap for the usual care and intervention phases, indicating little difference in event rates over time. Two exceptions are for in-hospital deaths and re-admissions. For deaths, the probability is greater in Hospitals Y and Z during the intervention phase. For readmissions, there is a slower accumulation of readmissions during the intervention phase in Hospital Y and greater number of readmissions after day 30 during the intervention phase in Hospital Z.

There was a protocol deviation during the trial as one clinical team did not receive the automated emails about at-risk patients due to a programming error, meaning they did not receive the full intervention. In a per-protocol analysis, we analysed this team as if it remained in the usual care phase for the whole trial; the results were similar to the intention-to-treat analysis (Appendix 4).

## Discussion

Most patients screened in this study were found to be nearing the end of their life and at risk of non-beneficial treatment. It was disappointing that the intervention showed no effect on avoiding ICU admissions as the primary outcome. Admission to the ICU was assumed to indicate a potential escalation of non-beneficial treatment in view of a poor prognosis. However, sometimes patients are admitted to the ICU on a ‘time trial’ basis where a potentially straightforward problem is suspected, which can be readily reversed, and they can be returned to the general ward rapidly. A medical emergency team call is possibly a more accurate outcome for non-beneficial treatment as the patient has presumably been receiving active management and not identified as being seriously ill until the last few hours/days of life [8, 36, 37]. However, we found no difference in the time to first medical emergency team call during the intervention phase, despite the awareness raising of the risk-of-death flag.

Most secondary outcomes also showed no benefit with the intervention, but there was a reduction in re-admissions to hospital in the intervention phase. This could have occurred because intervention patients were discharged with better supportive and palliative care plans in place. Hence, if their health again deteriorated, rather than coming to hospital they may have remained at home, including in an aged care facility. However, our previous analysis showed no or negative changes on care directive review activities including specialist palliative care referrals [16]. The fewer hospital re-admissions could also be related to the increase in deaths during the intervention; if the very sickest patients died, then there could be an overall reduced risk of re-admission in the remaining patients discharged alive. The estimated 0.06 reduction in probability of re-admission (Table 2) is relatively small at the individual level. Still, this reduction could have practical significance given the costs of admissions and the benefits to individuals wanting to die at home.

Our study assumed that physicians in the acute hospitals had full responsibility for the care of patients admitted under them. The physicians came from a variety of medical and surgical specialties working in three large teaching hospitals. The hypothesis was that if they were informed of a prognosis consistent with nearing end of life, their management would change, including admission to the ICU. This did not happen. A further study engaging specialist geriatricians in the management of these patients is worth considering and may result in a better outcome.

Overall, the trial provides no evidence to support widespread adoption of the single-component feedback loop InterACT intervention. A previous intervention that flagged patients’ likelihood of 6-month survival with clinicians also failed to improve care or patient outcomes [38]. Rebalancing death and dying will require large changes in hospital systems [15]. The ‘nudge’ intervention trialled here may not have been radical enough to change practice, and stronger, more directive, interventions alongside reminders and hospital policy changes may be needed. A more effective intervention may be for nurses or social workers to start the end-of-life discussions with the patients and their families rather than nudging doctors (or patients, families and clinicians together), as some families may be the best advocates for their older relatives and may not realise when they are nearing the end of life. However, hospital-based interventions may not be ideal for many families when their relative is acutely ill. A study of conversations between surrogates and clinicians in ICUs in the USA found little deliberation about the patient’s values and preferences [39]. Conversations to inform shared decision-making would ideally begin before people were admitted to hospital. General conversations about death and dying need to be normalised [15].

A potentially important explanation for why the intervention was unsuccessful is that the flags for at-risk patients were sometimes received too late, after decisions about patient care had already been made. A manual chart review process was required to collate the data needed for the CriSTAL and SPICT™ tools, and the study team only conducted the screening on two working days per week. A fully automated system could provide more timely information [40], but the data needed to complete the CriSTAL and SPICT™ tools were not routinely available in the study hospitals, and an automated system would require changes to hospital IT systems that are often administratively difficult.

Despite the delay in flags, initial treatment decisions could have been updated once patients were flagged at-risk. However, staff may have been unwilling to change existing care plans developed by senior staff, and a related study identified a ‘bystander’ effect with a diffusion of responsibility around end-of-life decision-making [41]. The same study found that prognostic tools were not commonly used to identify patients at risk of death, and instead, clinicians identified signs of dying using a combination of analytical, methodical thinking, intuition and pattern matching [41].

The process evaluation, which included interviews with clinicians enrolled in the trial, revealed that some clinicians felt able to identify patients near the end of life and questioned the sensitivity of the screening tools; specifically, two medical teams from one hospital found that the at-risk score of two for the SPICT tool was achieved by the vast majority of their patients as they all received one point for an unplanned admission [17]. The interviews also found ‘death denying’ views amongst some clinicians and a perceived lack of educational preparation in end-of-life care.

The strong variation between hospitals for some outcomes (Figure 3) suggests that it may not be appropriate to generalise the trial results to other large tertiary hospitals. The variation in outcomes could be due to: the complexity of end-of-life care; institutional resources, local policies and culture; and heterogeneity of local health information systems.

### Limitations

Stepped-wedge trials are vulnerable to confounding by other changes over time compared with parallel randomised trials. Important changes over time in this trial were the seasonal changes in the outcomes and the COVID-19 pandemic. The pandemic created public hesitancy around presenting to emergency departments, with an almost 20% decline in presentations in Queensland during the lockdown phase (March to June 2020) [42] and increased use of telemedicine [43, 44]. This decline overlapped with the start of the trial and may have decreased re-admissions and shortened lengths of stay during the usual care phase in all three hospitals as some introduced early discharge practices in preparation for the anticipated surge in COVID-19-related admissions. A qualitative study in the Netherlands found that the high clinical workload and social distancing due to the pandemic meant less time for end-of-life conversations between clinicians and families [45], and a related study in Australia found that hospital staff often found it more difficult to communicate with families because of reduced face-to-face interactions [46]. However, the pandemic also resulted in increased awareness of the need for advance care planning [46, 47] in part due to the often highly medicalised deaths suffered by older people who died alone in hospital [15].

We statistically adjusted for the seasonal changes in outcomes, but adjustment by design is always preferable to statistical adjustment. Future stepped-wedge or other cluster-randomised trials could reduce seasonal confounding by shortening the data collection periods and increasing the number of clusters. The staircase cluster randomised trial design [48] is a variation of the stepped wedge design, where data are collected for a limited number of time steps immediately before and after intervention exposure. However, these alternate designs focus on the immediate effects of the intervention at the expense of learning about long-term effectiveness. Researchers should also consider whether the intervention could be randomised at a patient level as parallel randomised trials cannot be confounded by season and some system-level changes may not require a clustered design.

Our power calculation assumed a proportion of ICU admissions of 0.20 for usual care patients, which was far higher than the observed proportion of 0.015. This occurred because we used a study that examined futile care in patients who had died [5], meaning our sample size was based on a group who were generally sicker than the target sample. Using this high expected proportion of patients meant we were over-powered to find the same-sized absolute difference for the primary outcome.

We did not formally adjust for multiple comparisons as we prefer to state what statistical comparisons we made and why, and have avoided ‘bright-line’ interpretations concerning statistical significance [49]. However, we acknowledge that the trial involved multiple secondary outcomes, and the one beneficial association of fewer re-admissions should be interpreted with caution. A cost-consequence study is underway, and this will summarise the overall benefits of the intervention using all outcomes combined, together with the costs incurred and saved.

## Conclusion

This nudge intervention to enhance awareness of patients’ risk of death with a single notification to doctors failed to demonstrate impact on reduction of ICU admissions, time to discharge and time to first MET call. Delayed notification of the at-risk flag to the treating team, high in-hospital mortality, changes in admission patterns and discharge practices during the COVID-19 pandemic may have contributed to the failure of the intervention. Additional reinforcements to the at-risk flag and earlier discussions with patients and their family advocates may be needed to achieve higher awareness of the benefits of reduction in non-beneficial treatments for patients near the end of life.

## Supplementary Material

aa-23-1695-File002_afae115

aa-23-1695-File003_afae115

aa-23-1695-File004_afae115

aa-23-1695-File005_afae115
