# Supplementary material for: Impact of a prospective feedback loop aimed at reducing non-beneficial treatments in older people admitted to hospital and potentially nearing the end of life. A cluster stepped-wedge randomised controlled trial
Source: Age Ageing. 2024 Jun 9;53(6):afae115. doi: 10.1093/ageing/afae115 (PMC11162291; doi:10.1093/ageing/afae115)
Supplement: aa-23-1695-File002_afae115 [file aa-23-1695-file002_afae115.docx]

## Appendix 1. Stepped-wedge study design diagram.


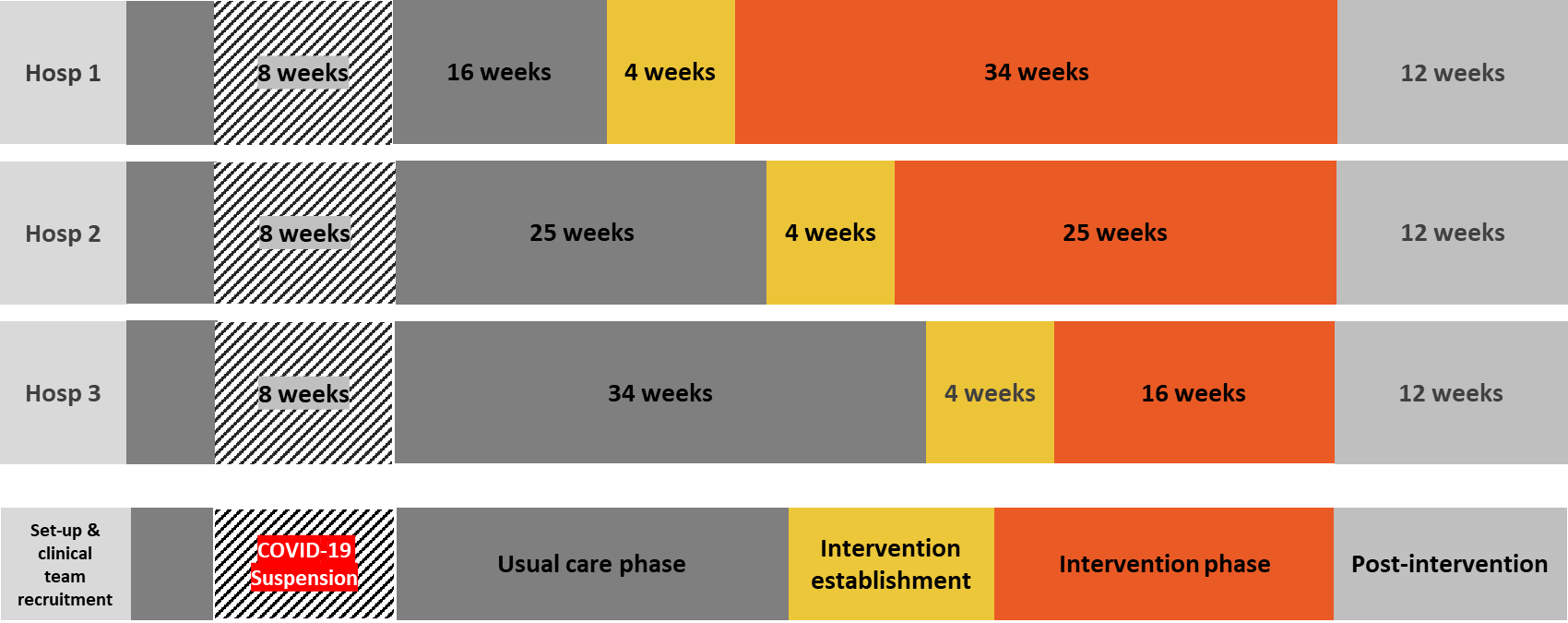


Figure A.1. Stepped-wedge design showing the usual care, intervention establishment, and intervention phases in the three hospitals. The post-intervention phase was used to collect interview data from clinical teams and the hospitals’ advisory groups.
