# Supplementary material for: Impact of a prospective feedback loop aimed at reducing non-beneficial treatments in older people admitted to hospital and potentially nearing the end of life. A cluster stepped-wedge randomised controlled trial
Source: Age Ageing. 2024 Jun 9;53(6):afae115. doi: 10.1093/ageing/afae115 (PMC11162291; doi:10.1093/ageing/afae115)
Supplement: aa-23-1695-File003_afae115 [file aa-23-1695-file003_afae115.docx]

## Appendix 2. Example report for an individual at-risk patient emailed to the treating doctor.


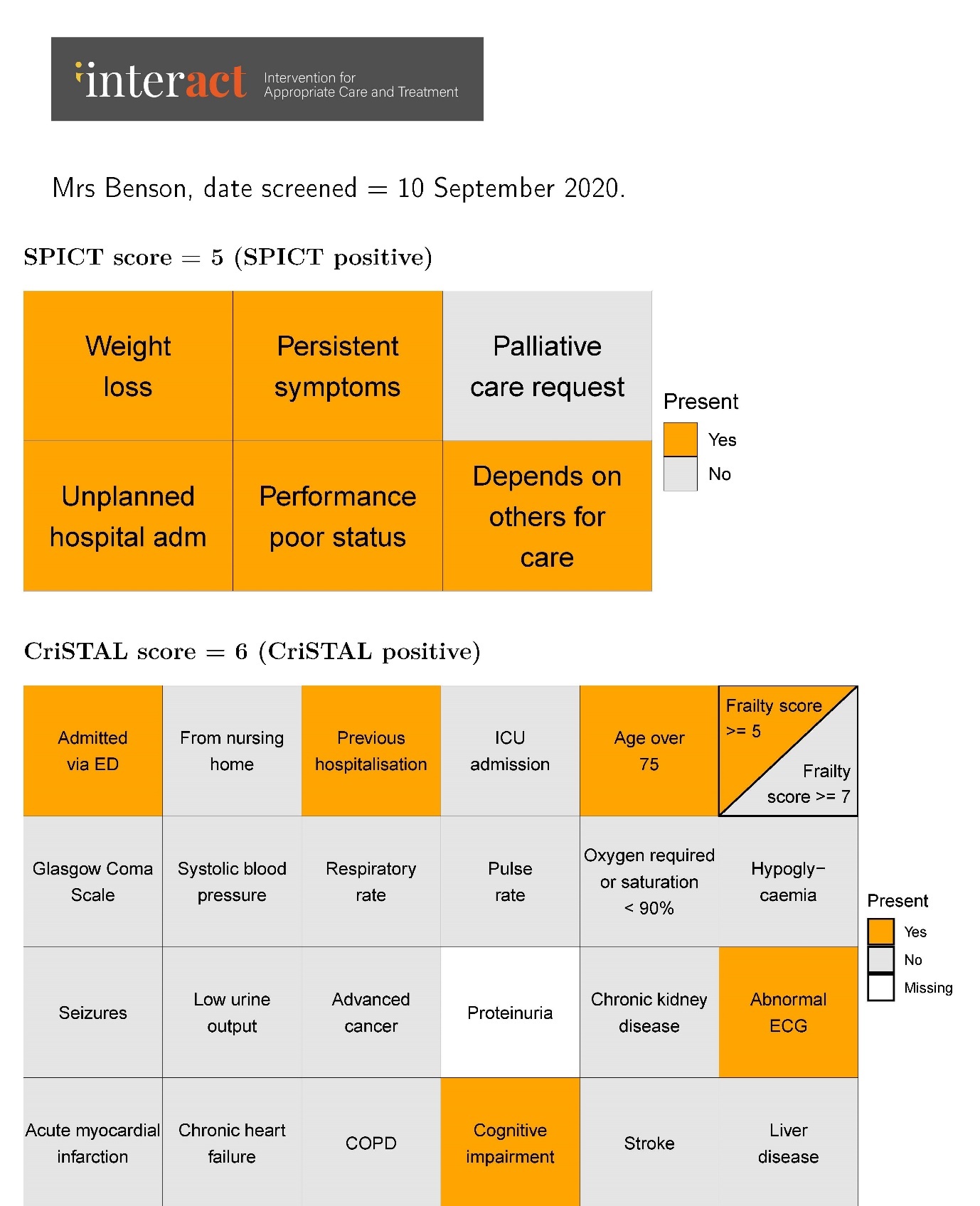


The patient’s name and data shown above are fictional.
