# Supplementary material for: Impact of a prospective feedback loop aimed at reducing non-beneficial treatments in older people admitted to hospital and potentially nearing the end of life. A cluster stepped-wedge randomised controlled trial
Source: Age Ageing. 2024 Jun 9;53(6):afae115. doi: 10.1093/ageing/afae115 (PMC11162291; doi:10.1093/ageing/afae115)
Supplement: aa-23-1695-File004_afae115 [file aa-23-1695-file004_afae115.docx]

## Appendix 3. Details on the statistical models

The primary outcome of ICU admission was modelled as:

$$Y_{i}\sim\text{Bernoulli}\left( \pi_{i} \right),\begin{matrix} & i=1, \ldots, N, \end{matrix}$$

$$\text{logit}\left( \pi_{i} \right)=\alpha_{0}+\sum_{j=1}^{4} \alpha_{j}x_{i,j}+\gamma_{j\left( i \right)},$$

$$\gamma_{j}\sim N\left( 0,\sigma^{2} \right),\begin{matrix} & j=1, \ldots,M \end{matrix},$$

where *Y_i_* is the binary outcome of ICU admission or not for admission *i*, *N* is the total number of at-risk admissions, **x** is a *N* × 4 matrix of independent variables of: the binary indicator for intervention phase (based on patient’s admission date), study week to account for a linear time trend, patient’s gender, and patient’s age. Age was centred at 85 and scaled by 5 years. Expected differences between the *M* treating teams were modelled using a random intercept (**γ**).

In a sensitivity analysis we added a seasonal effect to the regression equation which became

$$\text{logit}\left( \pi_{i} \right)=\alpha_{0}+\sum_{j=1}^{4} \alpha_{j}x_{i,j}+\beta_{1}\cos\left( \frac{2{\pi d}_{i}}{365.25} \right)+\beta_{2}\sin\left( \frac{2\pi d_{i}}{365.25} \right)+\gamma_{j\left( i \right)},$$

where *d_i_* is the admission date expressed as days. This seasonal effect is an annual sinusoid (Barnett & Dabson, 2010).

For time to event outcomes, we used a Cox model with strata for clinical teams so each team has its own baseline hazard which adjusted for differences between teams. We used separate Cox models for times to death and discharge as they were competing risks. We examined the time to the first event rather than the total number of events, for example, the time to first medical emergency team call rather than the total number of calls during the patient’s hospital admission. This is because the first event is often crucial and potentially sets patients down a very different care pathway. The total count of events per patients may not be independent and hence would be difficult to model as a Poisson process.

Censoring was used in the time to event analyses to avoid contamination of the estimated intervention effect from patients exposed to multiple study periods as follows:

- Data collected in the intervention establishment phase were excluded. Patients who were not discharged at the change-over time to the establishment phase were censored on the day prior to the change-over.
- Patients who were not discharged at the end of the intervention phase were censored on the last study day.

We used graphical model checks to assess the model assumptions. We plotted influential observations using the DFBETA statistic against observation number (Barnett & Dabson, 2010). We plotted the baseline cumulative hazard functions for the Cox models to identify outlying clinical teams. These results are available here: <https://github.com/agbarnett/InterACT/data_linkage> in the file “5_outcome_analysis.docx”.
