# Supplementary material for: Impact of a prospective feedback loop aimed at reducing non-beneficial treatments in older people admitted to hospital and potentially nearing the end of life. A cluster stepped-wedge randomised controlled trial
Source: Age Ageing. 2024 Jun 9;53(6):afae115. doi: 10.1093/ageing/afae115 (PMC11162291; doi:10.1093/ageing/afae115)
Supplement: aa-23-1695-File005_afae115 [file aa-23-1695-file005_afae115.docx]

Appendix 4. Protocol deviation

One clinical team did not receive any emails (second notification) during the intervention phase due to a programming error in our automated system. This team therefore did not receive the full intervention. Hence in an unplanned sensitivity analysis, this team had their intervention phase data included as part of the usual care phase (per protocol analysis). The original analysis is an “intention-to-treat” as we analysed the clinical team as they were randomised. The error occurred in one of the smaller teams and the per protocol and intention-to-treat results are similar:

|  |  | **Intention-to-treat** | **Per protocol** |
| --- | --- | --- | --- |
| **Outcome** | **Estimate** | **Mean and 95% CI** | **Mean and 95% CI** |
| ICU admission | Odds ratio | 0.84 (0.33 to 2.16) | 0.81 (0.34 to 1.94) |
| Time to discharged alive | Hazard ratio | 1.01 (0.94 to 1.08) | 1.02 (0.95 to 1.10) |
| Time to death in hospital | Hazard ratio | 1.36 (1.06 to 1.75) | 1.36 (1.05 to 1.75) |
| Time to medical emergency call | Hazard ratio | 0.93 (0.75 to 1.15) | 0.95 (0.76 to 1.19) |
| Time to hospital re-admission | Hazard ratio | 0.84 (0.76 to 0.93) | 0.86 (0.77 to 0.96) |
